# Evolution of C2H2-zinc finger genes revisited

**DOI:** 10.1186/1471-2148-9-51

**Published:** 2009-03-04

**Authors:** James H Thomas, Ryan O Emerson

**Affiliations:** 1 Department of Genome Sciences, University of Washington, Seattle, WA 91895, USA

## Abstract

**Background:**

A recent study by Tadepally et al. describes the clustering of zinc finger (ZF) genes in the human genome and traces their evolutionary history among several placental mammals with complete or draft genome sequences. One of the main conclusions from the paper is that there is a dramatic rate of gene duplication and gene loss, including the surprising result that 118 human ZF genes are absent in chimpanzee. The authors also present evidence concerning the ancestral order in which the ZF-associated KRAB and SCAN domains were recruited to ZF proteins.

**Results:**

Based on our analysis of two of the largest human ZF gene clusters, we find that nearly all of the human genes have plausible orthologs in chimpanzee. The one exception may be a result of the incomplete sequence coverage in the draft chimpanzee genome. The discrepancy in gene content analysis may result from the authors' dependence on the preliminary NCBI gene prediction set for chimpanzee, which appears to either fail to predict or to mispredict many chimpanzee ZF genes. Similar problems may affect the authors' interpretation of the more divergent dog, mouse, and rat ZF gene complements. In addition, we present evidence that the KRAB domain was recruited to ZF genes before the SCAN domain, rather than the reverse as the authors suggest. This discrepancy appears to result from the fact that the SCAN domain did indeed arise before the KRAB domain but is present only in non-ZF genes until a much later date.

**Conclusion:**

When comparing gene content among species, especially when using draft genome assemblies, dependence on preliminary gene prediction sets can be seriously misleading. In such studies, genic sequences must be identified in a manner that is as independent as possible of prediction sets. In addition, we present evidence that provides a more parsimonious explanation for the large proportion of mammalian KRAB-ZF genes without a SCAN domain.

## Background

The class of genes encoding multiple zinc finger (ZF) domains is very large in mammals and appears to have expanded on the primate lineage (e.g. [1]). An accurate picture of patterns of gene duplication and divergence and gene loss is critical for understanding evolution in this gene superfamily. The ZF gene complement in the human genome is fairly well established as a result of intensive EST and mRNA sequencing in humans and detailed hand-curation of a major part of the ZF superfamily [1]. All other mammalian genomes currently lack both of these advantages, and yet preliminary gene prediction sets are widely available, for example at NCBI and Ensembl  and . In the ZF superfamily, our unpublished studies show that these predictions frequently miss genes entirely or are obviously incorrect. In the recent paper by Tadepally et al. [2], we were struck by some remarkable claims about gene content in the ZF family. Among these claims are the complete absence of 118 human ZF genes in the chimpanzee genome (about 23% of all ZF genes) and some dog ZF gene predictions encoding huge numbers of ZF domains. Further investigation showed that the results in the paper appear to be based on available ZF gene predictions, and that these predictions are badly flawed.

Over half of the ZF genes in the human genome also contain an N-terminal KRAB or SCAN domain, or both. The KRAB domain is tetrapod-specific [3] and is nearly always found in ZF proteins. The SCAN domain has a broad distribution in vertebrates and is sometimes present in non-ZF proteins [4]. Tadepally et al. [2] present evidence that addition of the SCAN domain to ZF genes occurred first, followed later by addition of the KRAB domain to a SCAN-ZF gene. Since the large majority of mammalian KRAB-ZF genes lack a SCAN domain, this inference requires that the SCAN domain was secondarily lost in most genes.

## Results and discussion

### ZF orthologs in chimpanzee

**Figure 1:**
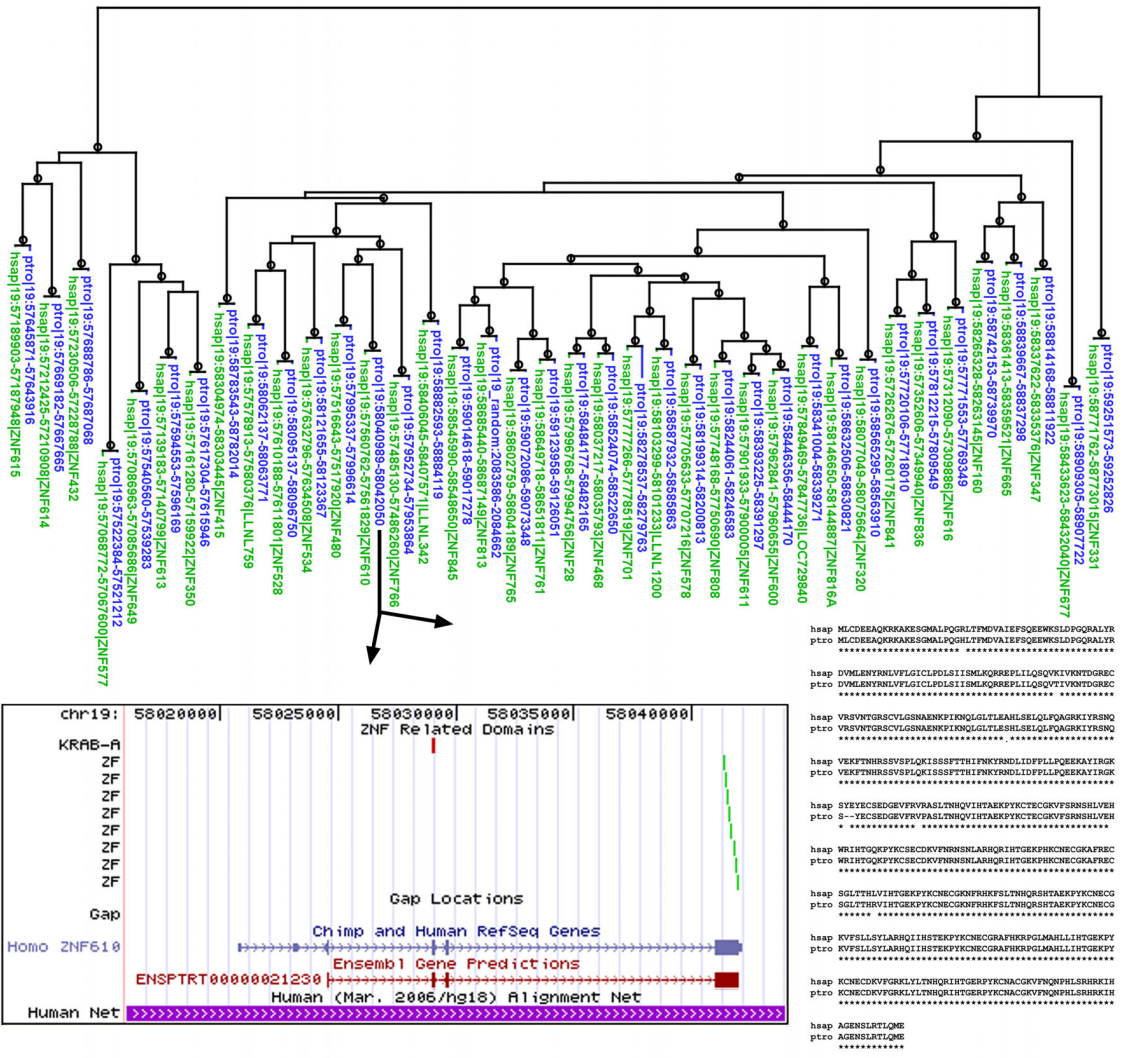
**The top panel shows a maximum-likelihood tree for all the human proteins (green) encoded in cluster 19.12 and their putative chimpanzee orthologs (blue)**. Black circles on branches indicate aLRT branch support of 0.95 or higher. The groups from Figure 5 of Tadepally et al. [2] correspond to the leftmost seven pairs of proteins (group III), the rightmost single pair of proteins (group I), and the rest of the tree (group II). Only the ZF exon regions were used in constructing the tree (see Methods). The genome position of each ZF exon is given in the fasta name. The lower left panel shows a UCSC browser image for 28 kb around chimpanzee ZNF610, one of the genes reported as absent from chimpanzee by Tadepally et al. [2]. The track "ZNF Related Domains" shows genomic domain matches from our added track (Additional file 2); the track "Gap Locations" shows the absence of known sequence gaps in this region; the RefSeq track shows the standard UCSC alignment of human ZNF610 to the chimpanzee genome; the Ensembl track shows an Ensembl gene prediction for the chimpanzee ZNF610 ortholog; the Human Net track shows that the entire region is syntenic to human chr 19. The lower right panel shows the chimpanzee protein aligned to its human ZNF610 ortholog.

Tadepally et al. [2] report that 118 of 510 human ZF genes are missing in the chimpanzee genome assembly and that many others are pseudogenes. Given the generally high conservation between human and chimpanzee, this is an extremely surprising result [5]. We investigated this pattern for two of the large ZF gene clusters on chromosome 19, including the gene cluster analyzed in detail in their Figure Five. Using their nomenclature, these are cluster 19.6 (28 human genes, 9 reported as missing in chimpanzee and another five reported as pseudogenes) and cluster 19.12 (43 human genes, 14 reported as missing in chimpanzee and another 10 reported as pseudogenes). According to our analysis, nearly all of the human proteins in these clusters contain a KRAB domain (see Methods and Additional file 1). The KRAB containing subset of the ZF superfamily has been carefully hand-curated in the human genome [1]. Nearly all KZNF (KRAB-zinc finger) genes in humans have a similar exon structure: the entire set of ZF domains is encoded on a single long 3' exon and the KRAB domain is split among one or more short 5' exons [1]. Typically, 80–90% of the final protein is encoded by the ZF exon. We took advantage of this fact to identify putative chimpanzee genes based on a simple tblastn method, which works independently of gene predictions. Combining results from the two gene clusters, we identified probable chimpanzee orthologs for 69 of 70 human KZNF genes (the slight change in human gene count results from current database predictions of 5 new ZF genes and retirement of 6 ZF genes as probable pseudogenes, see Additional file 1). It is possible that the single missing gene is a result of incomplete sequence coverage in the current chimpanzee genome assembly. Our results for cluster 19.12 are summarized in Figure 1 and tabular results for both clusters are given in Additional file 1, including predicted domain content and genome positions of the putative chimpanzee genes. Candidate orthologs for nearly all the human genes were also found in the orangutan genome but were not analyzed in detail (data not shown). The 69 putative chimpanzee orthologs were identified based only on their ZF-encoding exon. To test whether these exons are plausibly part of full KRAB-containing ZF genes, we used tblastn to search for ORFs that encode the most conserved part of the KRAB domain. 64 of the 69 putative chimpanzee ZF exons had a potential KRAB-encoding exon within 13 kb upstream (mean 7.0 kb). Most of the chimpanzee predictions that lacked a nearby KRAB exon correspond to human genes that are also predicted to lack a KRAB domain (Additional file 1). We also derived full gene models for 11 of the chimpanzee genes from cluster 19.12, nine of which are given as missing in chimpanzee in Tadepally et al. [2]. All 11 predictions aligned with their human counterparts with only a few amino acid changes; one is shown in Figure 1. Finally, Ensembl chimpanzee gene predictions exist for 14 of the genes from cluster 19.12 given as missing or defective in Tadepally et al. [2]. Though we remain uncertain about how many of the 69 putative chimpanzee genes from these two gene clusters will prove to be functional, our results are in much better accord with the high degree of overall similarity of the human and chimpanzee genomes. At the very least, sequence corresponding to no more than one of the 70 human genes is entirely missing in chimpanzee. We conclude that it remains possible that there is a perfect one-to-one correspondence of ZF genes between human and chimpanzee. To facilitate viewing potential ZF genes in the chimpanzee genome, we conducted a whole genome profile search and compiled the results as a BED format text file that can be loaded into the UCSC genome browser (see Methods and Additional file 2). The positions of all genomic matches to common ZF-associated domains (KRAB, SCAN, ZF, SET, and BTB) appear in this track regardless of prediction status. With this track displayed in full, inspection of the chimpanzee genome in the regions corresponding to human ZF gene clusters reveals multiple potential ZF genes that are currently unpredicted and unannotated.

We used the same methods in a global search for putative chimpanzee orthologs to the ZF exon from all 367 human KZNF genes with good experimental support. Strong candidate orthologs were identified for 343 of 367 genes and incompletely assembled orthologs were identified for another 18 genes (see Methods). The human and chimpanzee proteins encoded by all of these ZF exons are provided in Additional file 3, including genome coordinates and putative orthology assignments.

### Zinc finger counts and other genomes

Tadepally et al. [2] also report the presence of a ZF gene in the dog genome that encodes 70 tandem zinc fingers, far more than in any known human gene. Further investigation indicated that a number of other dog predictions similarly included unusually large numbers of ZF domains (data not shown). Inspection and analysis of the corresponding gene predictions in Entrez gene reports showed that these predictions included two or more large ZF-encoding exons . For example, the 70 ZF prediction (LOC484264) spans a 200 kb genomic region that includes 9 long ZF-encoding ORFs, 7 on the minus strand and 2 on the plus strand (Additional file 4). Parts of most of the minus strand ORFs are incorporated into the 70 ZF prediction, joined by long introns. Genome searches with the KRAB domain revealed that all 9 ZF-encoding ORFs in the region have a potential KRAB-containing ORF close upstream (mean distance 4.1 kb). Finally, two of the ZF ORFs appear to be orthologous to two human KZNF genes (one from each strand in the dog genome, data not shown). We conclude that the single LOC484264 prediction spans a region that likely encodes 9 distinct KZNF genes. Though this degree of inaccuracy in predictions is probably an extreme, it is clear that the dog prediction set cannot be used meaningfully to compare with other genomes. Similar problems are likely to affect the mouse and rat prediction sets (Additional file 5 and data not shown).

### KRAB and SCAN domains

**Figure 2:**
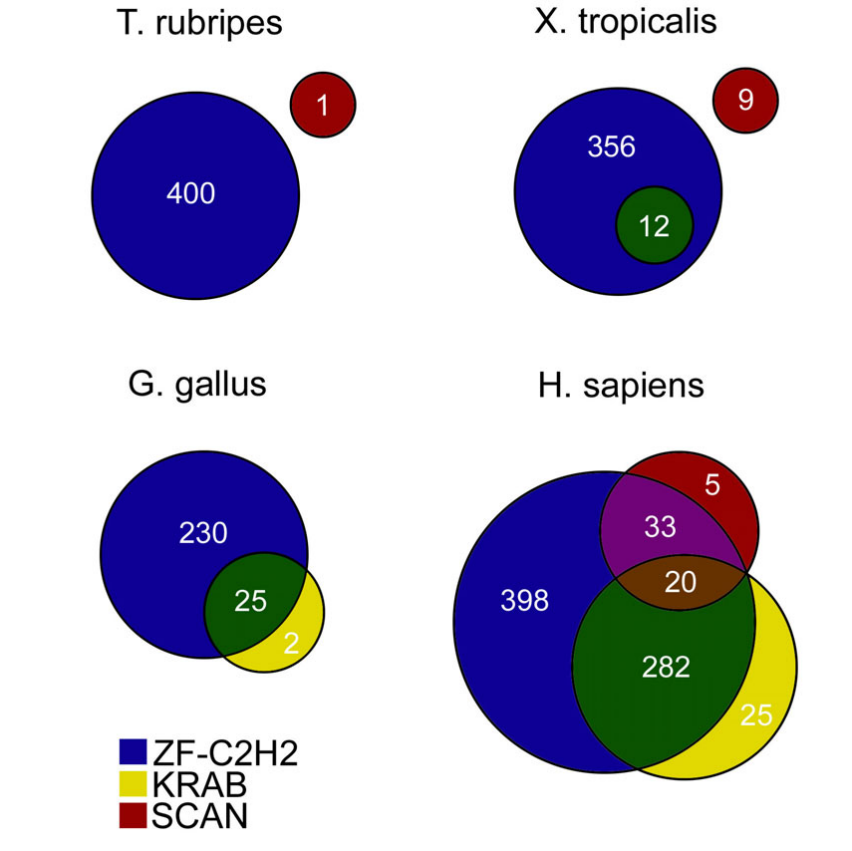
**For each of four species, the Venn diagram represents the number and overlap of predicted genes encoding ZF-C2H2 (blue), KRAB (yellow), and SCAN (red) domains**. Numbers correspond to the number of proteins in each category, e.g. chicken has 25 proteins with both KRAB and ZF-C2H2 domains and 2 proteins with only KRAB domains. SCAN domains are present in the prediction sets of *T. rubripes *and *X. tropicalis*, but are not associated with ZF-C2H2 proteins. The KRAB domain is not present in *T. rubripes*, but is already associated with ZF-C2H2 proteins in *X. tropicalis*. *H. sapiens *has broad overlaps in its sets of ZF-C2H2, KRAB and SCAN proteins, a pattern typical of mammals.

In order to assess the evolutionary history of the KRAB and SCAN domains with respect to the ZF gene family, we used HMMer software [6] to search all proteins in the Ensembl prediction sets for *Takifugu rubripes*, *Xenopus tropicalis*, *Gallus gallus *and *Homo sapiens*. Figure 2 shows the numbers and overlaps of proteins containing these three domains in each species. The SCAN domain is present in each species except for chicken, consistent with previous evidence suggesting that SCAN arose in the ancestor of vertebrates and has been lost in chicken [4,7,8]. In the fish and frog we analyzed, the SCAN domain is present but is not found in proteins that also contain ZF domains. This suggests that the fish, frog and chicken genomes contain no SCAN-ZF genes, although we cannot rule out unpredicted genes with this structure.

The KRAB domain is present in all three tetrapod species, *X. tropicalis*, *G. gallus *and *H. sapiens*, consistent with previous findings suggesting that the KRAB domain arose in the ancestor of tetrapod vertebrates [3,9]. Among the three species that contain KRAB domains, all three also contain many KRAB-ZF proteins. The large majority of predicted KRAB-containing proteins in chicken (25 of 27) and *X. tropicalis *(12 of 12) are KRAB-ZF proteins that lack a SCAN domain. We conclude that the association of the KRAB domain with ZF proteins arose in the ancestor of tetrapod vertebrates and therefore predates the association of the SCAN domain with ZF and KRAB-ZF proteins, which is observed only in mammals. This conclusion also parsimoniously explains why the large majority of human KRAB-ZF proteins do not have a SCAN domain.

## Conclusion

We show that two of the main conclusions of Tadepally et al. [2] are questionable. First, we show that the zinc finger gene content of chimpanzee was inadequately assessed, resulting in dramatic overestimates of the number of gene losses on the chimpanzee lineage following its divergence from human. Similar difficulties may apply to assessed gene contents of the mouse, dog, and rat genomes. Second, we show that the KRAB domain was probably added to zinc finger genes before the SCAN domain.

## Methods

### Gene identification and trees

Genomic sequences were collected from the current human and chimpanzee genome builds at UCSC (human reference sequence NCBI Build 36.1 and chimpanzee draft assembly Build 2 Version 1 (panTro2), both at ). The chimpanzee assembly is the same as that used by Tadepally et al. [2]; we also obtained identical results from the same chimpanzee assembly downloaded from Ensembl . Human ZF gene predictions for clusters 19.6 and 19.12 were derived by manual curation of predictions from NCBI RefSeq 25, ENSEMBL release 50, and the dedicated LLNL ZF annotation web resource [1]. We accepted the gene model that appeared to be full-length and that corresponded best to the canonical domain content and structural features of the gene family. The ZF domains of all genes were present on a single exon. We used the protein sequence from the ZF-encoding exon of each human gene as a tblastn query against the chimpanzee genome [10]. The DNA corresponding to each best match was extracted and its translation was validated as aligning well with its human query. In a few cases, a run of N residues or a stop codon interrupted the chimpanzee match; these were accepted as possible artifacts of an immature genome assembly and the remaining coding region was used in further analysis. The ZF exon protein sequence from all the human cluster 19.12 genes and their corresponding chimpanzee predictions were aligned using ClustalW (default parameters) [11]. From this multiple alignment, a maximum-likelihood (ML) tree with branch supports was made using phyml-alrt (JTT rate matrix, 6 rate categories, gamma-parameter 1.0) [12,13]. We note that this approach to analyzing the evolution of these rather diverse proteins is questionable, but we use this method to parallel Tadepally et al. Figure Five [2]. The main difficulty is in constructing a meaningful multiple alignment with proteins whose number of zinc fingers varies from 7 to 21. The validity of the ML tree based on the multiple alignment was cross-checked by making a pairwise distance clustering tree, which gave perfect agreement in ortholog assignment. Complete or nearly complete gene models for 11 of the chimpanzee genes corresponding to cluster 19.12 were made by using the entire human protein as query in a tblastn search, extracting the genomic DNA corresponding to the entire best match region, and manually curating splice junctions. Some of these predictions lack the first coding exon because it encoded only one or a few amino acids in the human and could not be identified with confidence.

We also used ZF exons from all 367 human KZNF genes with good experimental support in a tblastn search of the chimpanzee genome, extracted matching sequences, and used a pairwise distance clustering tree to test probable orthology. 344 of 367 ZF exons had strong candidate orthologs (at least 70% of coding sequence found). Of the 24 missing genes, 18 had shorter matches in the assembly or strong candidate matches (at least 200 amino acids of gap-free alignment with at least 98% amino acid identity) in the chimpanzee sequence trace archives , suggesting that these genes are present but partially assembled or unassembled in the chimpanzee genome. For 4 of these 18 we obtained matching reads and quality scores and made a local assembly of the ZF exon using the CAP3 assembler with default settings [14], confirming that the exon was present and aligned well with the human ortholog.

### Browser view of chimpanzee, dog, and mouse genomic profile matches

The 6-frame translating and tabular output options of rps-blast (-p F and -m 8 options) were used to locate all genome matches to common ZF-associated domains. The rps-blast program and the KRAB-A, C2H2 zinc finger, SCAN, BTB, and SET domain profiles were obtained from the NCBI download sites ( and ). E-values for the rps-blast search were very permissive to ensure that all potential domains were found. Inspection of the results in the UCSC browser indicated that there are very few false positives. The rps-blast output was converted to the UCSC BED format manually using Excel. The data in Additional files 3, 4 and 5 can be directly uploaded to the UCSC browser (assemblies chimpanzee panTro2, dog canFam2, and mouse mm9 respectively) to view the positions of all profile matches (use "add custom tracks" to load data). For example, dog position chr1:103615000-103820000 displays the region of the dubious 70 ZF prediction LOC484264, and clearly shows nine putative KRAB-ZF genes with the canonical structure of one KRAB-A exon close upstream of one long ZF exon.

### Existing prediction sets

A different way of viewing the inadequacy of current gene predictions came from analysis of the predicted gene content for cluster 19.12. Ten of the 29 chimpanzee NCBI genes given in Tadepally et al. Additional file 5[2] are annotated as pseudogenes with no protein prediction available (LOC468984, LOC468985, ZNF701, LOC456267, LOC456268, ZNF160, LOC456426, LOC456269, LOC456270, and ZNF468). Thus NCBI indicates only 19 predicted functional chimpanzee genes corresponding to human cluster 19.12, which has 43 human genes in Tadepally et al. [2]. Searches of Ensembl predicted genes identified 30 predicted functional chimpanzee genes corresponding to cluster 19.12. Sixteen of the Ensembl genes correspond to NCBI predictions, 14 are found only in Ensembl predictions, and three are found only in NCBI predictions.

### ZF protein domain content

We reassessed domain content for the predicted human ZF proteins from clusters 19.6 and 19.12. From an rps-blast search against the Pfam KRAB domain profile (PF01352) we found that 9 human proteins contained a KRAB domain not reported by Tadepally et al. [2], perhaps due to improved gene models. Since ZF profile searches identify abnormal ZF domains that are probably defective in binding DNA (for example many are missing one or more of the zinc coordinating residues), we used a regular expression pattern matcher to count canonical ZF domains in human and chimpanzee predictions, defined as those containing the canonical pattern C X_2 _C X_12 _H X_3 _H. KRAB and ZF domain content are given in Additional file 1.

### KRAB and SCAN domain origin

All protein sequences predicted from NCBI human build 36 were collected from Ensembl BioMart . Protein prediction sets were also collected from *Gallus gallus *WASHUC2, *Takifugu rubripes *FUGU4, and *Xenopus tropicalis *JGI4.1. HMMer software [6] and the Pfam database [15] were used to identify protein domain content. The Pfam profile of the C2H2-ZF domain (PF00096) was searched against each entire prediction set using HMMer version 2.3.2, and each protein with at least one hit to the profile with a permissive e-value (less than or equal to 0.1) was marked as a ZF-C2H2 protein. These searches were repeated with the KRAB (PF01352) and SCAN (PF02023) domain profiles. This procedure provides inclusive lists of predicted ZF, KRAB and SCAN proteins in each species. The highly divergent ancestral KRAB-like domain [3] was not found in these searches. Figure 2 represents the intersections of these lists in each of the four species considered.

## Authors' contributions

Both authors contributed equally. All authors read and approved the final manuscript.

## Response

By Hamsa Tadepally, Gertraud Burger and Muriel Aubry

Address: Department of Biochemistry, Université de Montréal, C.P 6128, Succ. Centre-Ville, Montréal QC H3C 3J7, Canada

Emails:

HT: Hamsa.tadepally@gmail.com

GB: Gertraud.burger@umontreal.ca

MA: Muriel.aubry@umontreal.ca

C2H2 zinc finger genes (C2H2-ZNF) constitute the largest class of transcription factors in humans and one of the largest gene families in mammals. The goal reached in our 2008 study [2] was to provide a first view of all the C2H2-zinc finger genes clustering in the human genome and of their syntenic counterparts in four other mammals, according to available genome assemblies, and a better insight into the evolution of this extremely large family of genes in mammals. We suggested that differential evolution of C2H2-ZNF genes occurred at the level of clusters, genes and effector domains in several mammals. This was in agreement with previous smaller scale studies focusing on specific chromosomes or sub-subfamilies of C2H2-ZNF in human, chimp and mouse [1,16-19]. This study provides an entry point for further large scale studies with an evolutionary perspective or designed to analyze the expression of clustering C2H2-ZNF genes in various tissues and species.

Thomas and Emerson report in their manuscript that the number of chimpanzee genes corresponding to two of the largest human C2H2-zinc finger (ZNF) clusters differs from what was originally mentioned in our study analyzing 81 clusters in five mammals [2]. Considering that our study was based on public databases where reliability keeps increasing with time for all genomes owing to regular updating, this possibility was foreseen as documented in our manuscript. More specifically, the discrepancy mentioned by Thomas and Emerson appears to originate from the fact that they used the chimpanzee draft assembly from UCSC  for their human-chimpanzee comparison whereas our large scale study of human C2H2 ZNF genes and syntenic counterparts in other mammals was performed using NCBI genome assemblies and data available from Ensembl at the time of publication (described clearly in Tadepally *et al.*[2]). In term of methods, as done by the Thomas'group, our prediction of the presence of zinc finger genes was based on TBLASTN searches of the chimpanzee regions which are syntenic to the human clusters (detailed in Tadepally et al[2]). Of note, as other highly repetitive regions in genomes, large clusters of zinc finger genes represents challenging regions of genomes difficult to assemble. The manuscript from Thomas and Emerson illustrates this by pointing out that the current chimpanzee draft assembly revealed clustered ZNF genes that were missing in the NCBI chimpanzee assembly at the time of publication of our study [2]; it thus has the merit to indicate that atypically large chromosome 19 human and chimpanzee C2H2-ZNF clusters, such as the two they examined, might be more similar than our study suggested based on available genomic data [2]. Considering that several studies suggested differential expansion of C2H2-ZNF genes in mammals [1,16-19], for better assessment of these differences it will be informative to determine if NCBI chimpanzee assemblies are more reliable for smaller clusters which are presumably easier to assemble and if other mammalian genome such as the mouse, rat and dog genome are presently more accurately assembled than the chimpanzee genome.

By reassessment of the two large C2H2-ZNF clusters discussed by Thomas and Emerson using the current NCBI 36 Version 3 human genome assembly (October 2008) rather than the one used at the time of our publication, we found that several genes previously annotated as pseudogenes are now described as genes or the reverse. This pattern was also noted in the NCBI chimpanzee genome assembly. This is in agreement with the data presented by Thomas and Emerson in their manuscript. We had pointed out in our study [2] that 'the percentage of C2H2-ZNF genes annotated as pseudogenes was higher in chimpanzee that in human C2H2-ZNF clusters'. Clearly, there are still changes to expect on the status of pseudogenes or genes for members of the C2H2-ZNF family in years to come based on the release of improved genome assemblies and detailed manual curation of individual genes for improved gene model and gene prediction. As a step, in that direction Thomas and Emerson re-evaluated the NCBI gene predictions by careful inspection of individual genes and provides evidence that in the dog genome, in particular, inaccurate predictions lead to the identification of a single gene in place of several through the erroneous joining of several zinc finger motif-containing exons that most likely belongs to tandemly organised zinc finger genes. This is based on the assumption that the zinc finger region of C2H2-ZNF genes should be encoded by a single exon as observed in the majority of the well documented cases with few exceptions [20,21], where the zinc finger region is encoded by more than one exon.

By analyzing the clusters described by Thomas and Emerson in their Supplemental data 1 (supp1.xls), we were struck by the fact that the reported number of zinc finger domains conforming to the consensus sequence were different from our results [2] and from those of the PFAM database. The classic consensus motif for zinc finger genes of the C2H2 type is: YE**C**X2-4**C**X3FX5LX2**H**X3-4**H**TGEKP as mentioned in Tadepally et al [2]. We noticed that the 'pattern' used for their analysis was 'CX2CX12HX3H'. In addition to eliminate most, if not all, the degenerate zinc finger motifs (eg. in ZNF443 which contains 4 degenerate zinc finger motifs out of 19 zinc finger motifs), the 'pattern' they chose has furthermore the drawback of missing all the zinc finger motifs with more than two or three amino acids in between the cysteines or the histidines, respectively. As an example, KLF1, a very well characterized member of the C2H2 zinc finger family with three zinc finger domains, that conform to the C2H2 consensus [22], is listed as 'divergent family member' in Supplemental data 1 of their manuscript and is reported to contain one zinc finger motif instead of three; two out of three bona fide zinc finger domains, conforming to the consensus C**X4**CX12H**X3**H, were not considered. In the study by Tadepally et al. [2], we have chosen to include degenerate zinc finger motifs in agreement with the PFAM database since, to our point of view, this represented an initial important information for comparison with other species and thus for evolutionary studies. The identification of the degenerate zinc finger motif (mainly based on the absence of one or several of the two cysteine and histidine residues) is however valuable information that could be compiled in further evolutionary studies.

In Tadepally et al. [2], we proposed a model of interdependent evolution of C2H2-ZNF gene subfamilies due to the deletion or degeneration of the SCAN or KRAB domain from SCAN, SCAN-KRAB or KRAB C2H2-ZNF subfamilies. In this model, we also suggested that singular and sequential gain of first a SCAN domain by a ZNF gene and then a KRAB domain by a SCAN-ZNF gene led to the appearance of the SCAN-ZNF and SCAN-KRAB-ZNF genes. This model was based on the sequence similarity and exon-intron patterns of these genes and what was previously known. This model remains to be fully evaluated. Thomas and Emerson provide evidence in favor of a revised model suggesting that the SCAN motif arose in ZNF gene after the KRAB domain. The model proposed by Thomas and Emerson is an interesting and quite possible way to look at the evolution of these genes. However, it would be interesting to see how their model incorporate the facts that i) SCAN C2H2-ZNF and SCAN-KRAB C2H2-ZNF have a specific conserved exon-intron pattern [2] and ii) SCAN-KRAB genes do not form a separate group in the phylogenetic trees of the KRAB domain [1].

In conclusion, our large scale study of over 700 C2H2-ZNF genes in human and on their syntenic counterparts in four other mammals provided a 2008 Polaroid picture for insight into the evolution of this interesting family of genes [2]. Adding to the already published literature on the evolution of C2H2-ZNF genes [1,2,16-19], complete curation of the various genomes, as stressed here by Thomas and Emerson, as well as sequencing of new genomes will provide more refined and accurate pictures of this amazingly large C2H2-ZNF gene family for better understanding of its evolution in various species and for studying the coordinated or differential regulation of the expression of these clustering genes.

## Supplementary Material

Additional file 1 **Supplemental Table 1.** Summary of the reanalysis of chimpanzee orthologs for human gene clusters 19.6 and 19.12. Click here for file

Additional file 2 **Supplemental Table 2.** Table of zinc-finger related profile matches for the entire chimpanzee genome. The table is in BED format and can be directly uploaded as a custom track on the UCSC genome browser. Click here for file

Additional file 3 **Supplemental Data 1.** Fasta protein sequences and genome positions corresponding to the ZF exon of 367 human KZNF genes and 344 putative chimpanzee orthologs. Click here for file

Additional file 4 **Supplemental Table 3. **Table of zinc-finger related profile matches for the entire dog genome. The table is in BED format and can be directly uploaded as a custom track on the UCSC genome browser. Click here for file

Additional file 5 **Supplemental Table 4.** Table of zinc-finger related profile matches for the entire mouse genome. The table is in BED format and can be directly uploaded as a custom track on the UCSC genome browser. Click here for file

## References

[B1] Huntley S, Baggott DM, Hamilton AT, Tran-Gyamfi M, Yang S, Kim J, Gordon L, Branscomb E, Stubbs L (2006). A comprehensive catalog of human KRAB-associated zinc finger genes: insights into the evolutionary history of a large family of transcriptional repressors. Genome Res.

[B2] Tadepally HD, Burger G, Aubry M (2008). Evolution of C2H2-zinc finger genes and subfamilies in mammals: species-specific duplication and loss of clusters, genes and effector domains. BMC Evol Biol.

[B3] Birtle Z, Ponting CP (2006). Meisetz and the birth of the KRAB motif. Bioinformatics.

[B4] Edelstein LC, Collins T (2005). The SCAN domain family of zinc finger transcription factors. Gene.

[B5] Chimpanzee Sequencing and Analysis Consortium (2005). Initial sequence of the chimpanzee genome and comparison with the human genome. Nature.

[B6] Eddy SR (1998). Profile hidden Markov models. Bioinformatics.

[B7] Williams AJ, Khachigian LM, Shows T, Collins T (1995). Isolation and characterization of a novel zinc-finger protein with transcription repressor activity. J Biol Chem.

[B8] Sander TL, Stringer KF, Maki JL, Szauter P, Stone JR, Collins T (2003). The SCAN domain defines a large family of zinc finger transcription factors. Gene.

[B9] Bellefroid EJ, Poncelet DA, Lecocq PJ, Revelant O, Martial JA (1991). The evolutionarily conserved Kruppel-associated box domain defines a subfamily of eukaryotic multifingered proteins. Proc Natl Acad Sci USA.

[B10] Altschul SF, Madden TL, Schaffer AA, Zhang J, Zhang Z, Miller W, Lipman DJ (1997). Gapped BLAST and PSI-BLAST: a new generation of protein database search programs. Nucleic Acids Res.

[B11] Thompson JD, Higgins DG, Gibson TJ (1994). CLUSTAL W: improving the sensitivity of progressive multiple sequence alignment through sequence weighting, position-specific gap penalties and weight matrix choice. Nucleic Acids Res.

[B12] Guindon S, Gascuel O (2003). A simple, fast, and accurate algorithm to estimate large phylogenies by maximum likelihood. Syst Biol.

[B13] Anisimova M, Gascuel O (2006). Approximate likelihood-ratio test for branches: A fast, accurate, and powerful alternative. Syst Biol.

[B14] Huang X, Madan A (1999). CAP3: A DNA sequence assembly program. Genome Res.

[B15] Bateman A, Birney E, Durbin R, Eddy SR, Howe KL, Sonnhammer EL (2000). The Pfam protein families database. Nucleic Acids Res.

[B16] Ding G, Lorenz P, Kreutzer M, Li Y, Thiesen HJ (2008). SysZNF: the C2H2 zinc finger gene database. Nucleic Acids Res.

[B17] Hamilton AT, Huntley S, Tran-Gyamfi M, Baggott DM, Gordon L, Stubbs L (2006). Evolutionary expansion and divergence in the ZNF91 subfamily of primate-specific zinc finger genes. Genome Res.

[B18] Looman C, Abrink M, Mark C, Hellman L (2002). KRAB zinc finger proteins: an analysis of the molecular mechanisms governing their increase in numbers and complexity during evolution. Mol Biol Evol.

[B19] Shannon M, Hamilton AT, Gordon L, Branscomb E, Stubbs L (2003). Differential expansion of zinc-finger transcription factor loci in homologous human and mouse gene clusters. Genome Res.

[B20] Kaczynski J, Cook T, Urrutia R (2003). Sp1- and Kruppel-like transcription factors. Genome Biol.

[B21] Urrutia R (2003). KRAB-containing zinc-finger repressor proteins. Genome Biol.

[B22] Eaton SA, Funnell AP, Sue N, Nicholas H, Pearson RC, Crossley M (2008). A network of Kruppel-like Factors (Klfs). Klf8 is repressed by Klf3 and activated by Klf1 in vivo. J Biol Chem.

